# Supplementary figures and images for: An interactive deep learning-based approach reveals mitochondrial cristae topologies
Source: PLoS Biol. 2023 Aug 31;21(8):e3002246. doi: 10.1371/journal.pbio.3002246 (PMC10470929; doi:10.1371/journal.pbio.3002246)

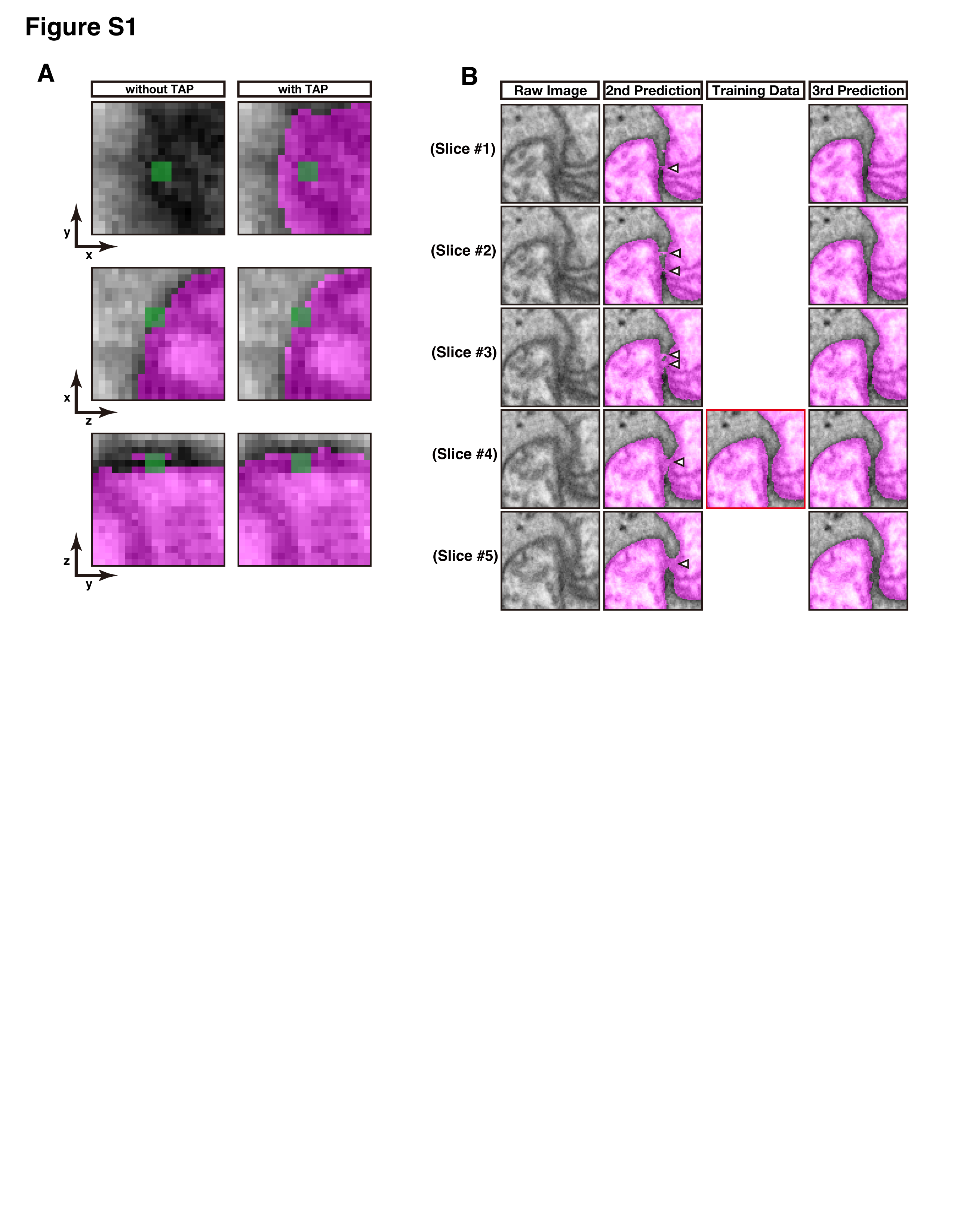

Supplement: S1 Fig — (A) Segmentations of a mitochondrion with or without the TAP method. Edges of a mitochondrion were not annotated as mitochondria (magenta) by a 2D UNet++ based prediction only from the xy-plane (without TAP). With the TAP method, the same edges were successfully annotated as mitochondria by combining the predictions from all 3 axes. The green square is a fiducial marker for correlating images from different axes. (B) Segmentation results of mitochondria closely apposed to each other (magenta) are shown. The prediction mistakenly annotated those mitochondria as connected in the second prediction (arrowheads). At the third prediction, by adding a training data corrected from the second prediction in slice #4, misannotation in other slices were also corrected. The raw EM data are deposited in the EMPIAR (EMPIAR-11449). EMPIAR, Electron Microscopy Public Image Archive; EM, electron microscopy; TAP, three-axes prediction; 2D, two-dimensional; HITL, human-in-the-loop. (TIF) [file pbio.3002246.s001.tif]

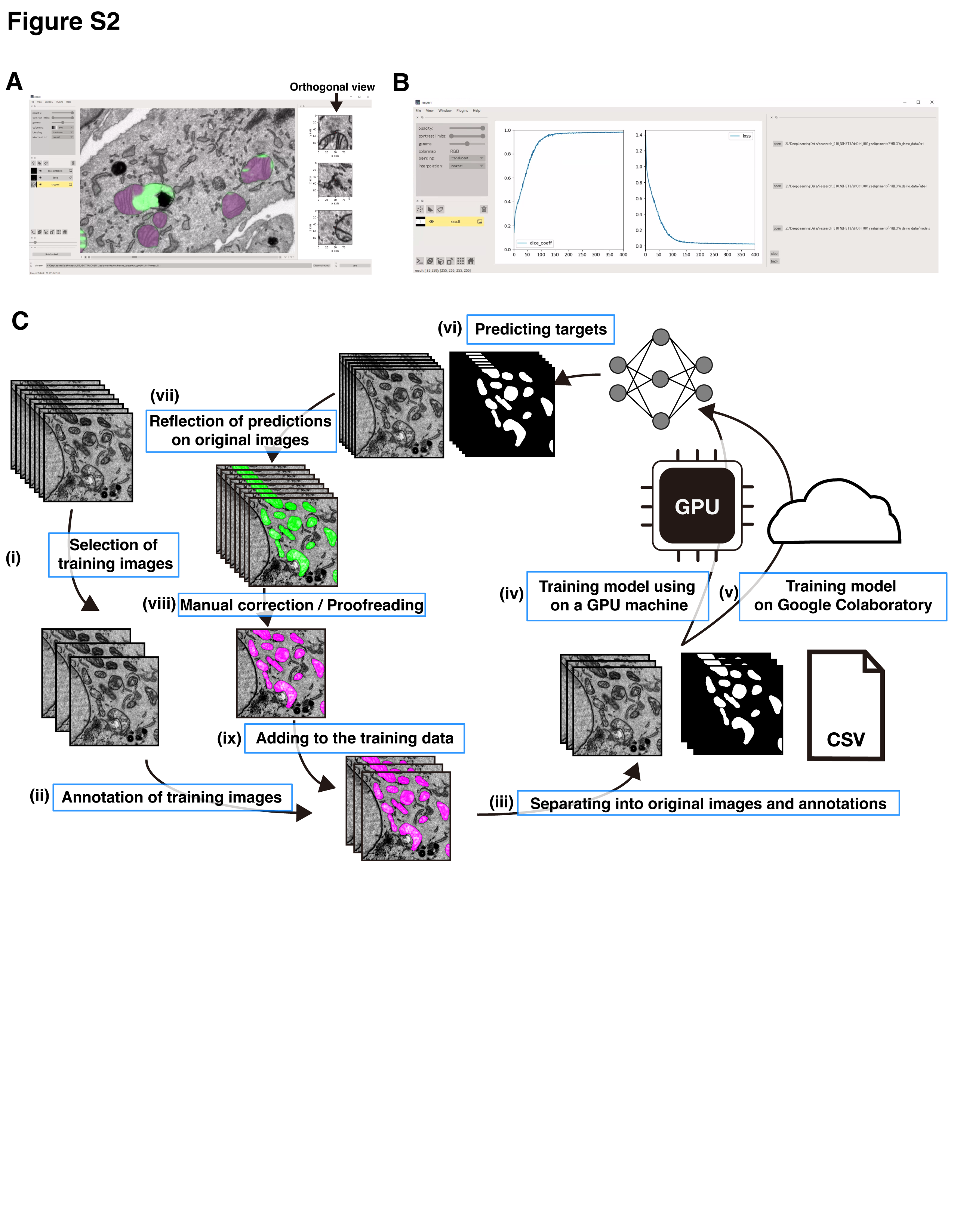

Supplement: S2 Fig — (A) GUI of PHILOW during annotation. The buttons for data management and the orthogonal view are shown. Low confidence areas are highlighted in green for active learning. (B) GUI of PHILOW showing the progress of a model training. The left graph shows the change of dice coefficient score along the number of epochs. The right graph shows the change of a loss function score along the number of epochs. By monitoring the score, users can stop the training at the moment the scores are saturated by clicking the stop button. (C) First, in the annotation mode, users select areas suitable for making training dataset by a button-click while observing the 3D data (i). Then, users annotate training images while looking at the orthogonal view in order to make accurate annotations (ii). The annotated images are automatically separated into original images and corresponding annotations for importing them into the model training mode (iii). The model training can be done either on a GPU machine via GUI (iv) or on Google Colaboratory (v). In the case of isotropic 3D data, our TAP method is applicable during inference, resulting in higher inference accuracy. The original images are applied to the trained model and predictions are generated (vi). The predictions are shown directly on the annotation mode for immediate proofreading (vii). The proofread slices (viii) can be added to the existing training data for the next training cycle (ix). After saturation of the prediction accuracy by iterative cycles, a final manual correction is also performed directly on PHILOW (see Fig 3A). The raw EM data are deposited in the EMPIAR (EMPIAR-11449). EMPIAR, Electron Microscopy Public Image Archive; EM, electron microscopy; GUI, graphical user interface; 3D, three-dimensional; GPU, graphics processing unit. (TIF) [file pbio.3002246.s002.tif]

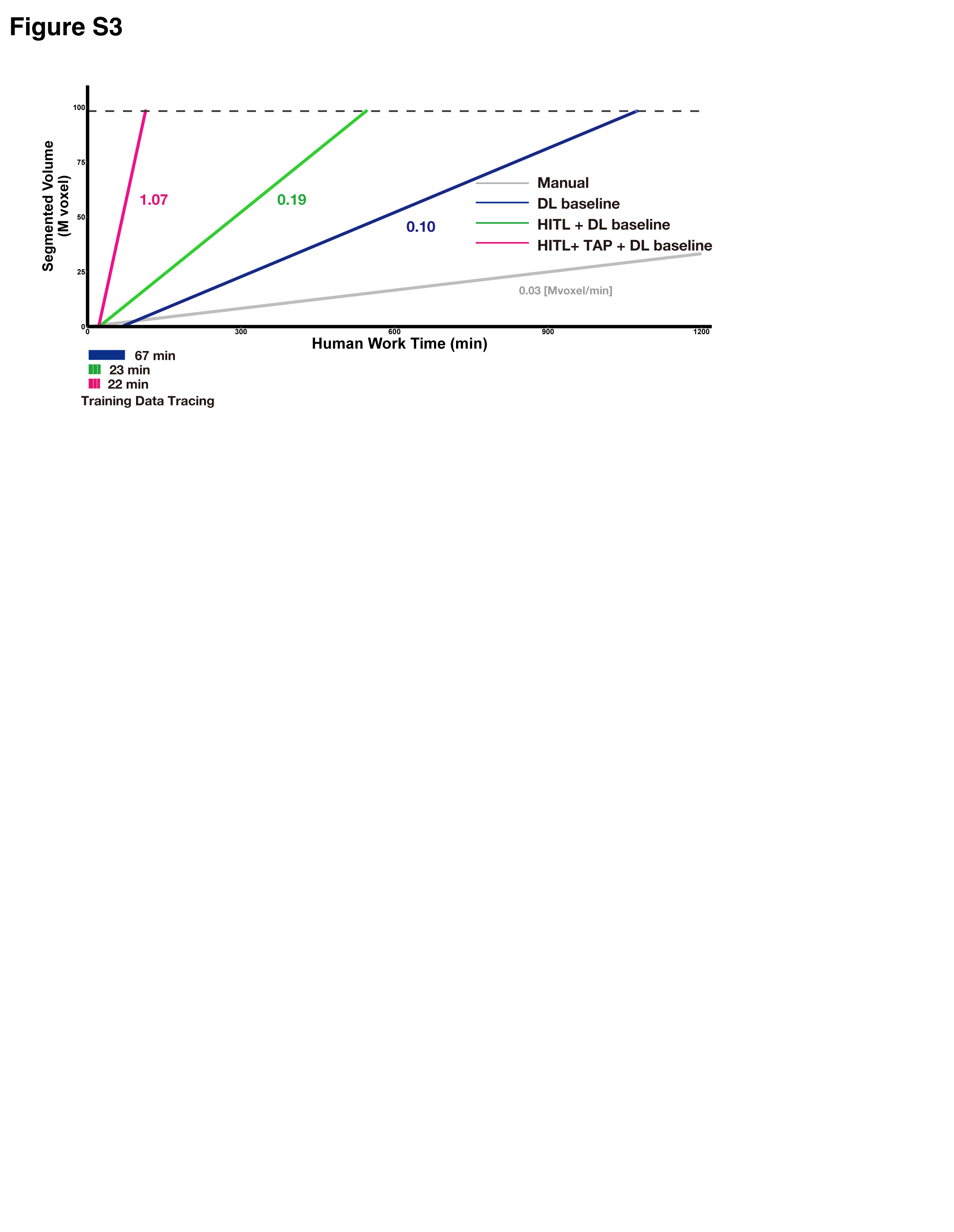

Supplement: S3 Fig — The same time measurement as Fig 3C conducted by another annotator. Source data can be found in S14 Data. The raw EM data are deposited in the EMPIAR (EMPIAR-11449). EMPIAR, Electron Microscopy Public Image Archive; EM, electron microscopy; HITL, human-in-the-loop; TAP, three-axes prediction; DL, deep learning. (TIF) [file pbio.3002246.s003.tif]

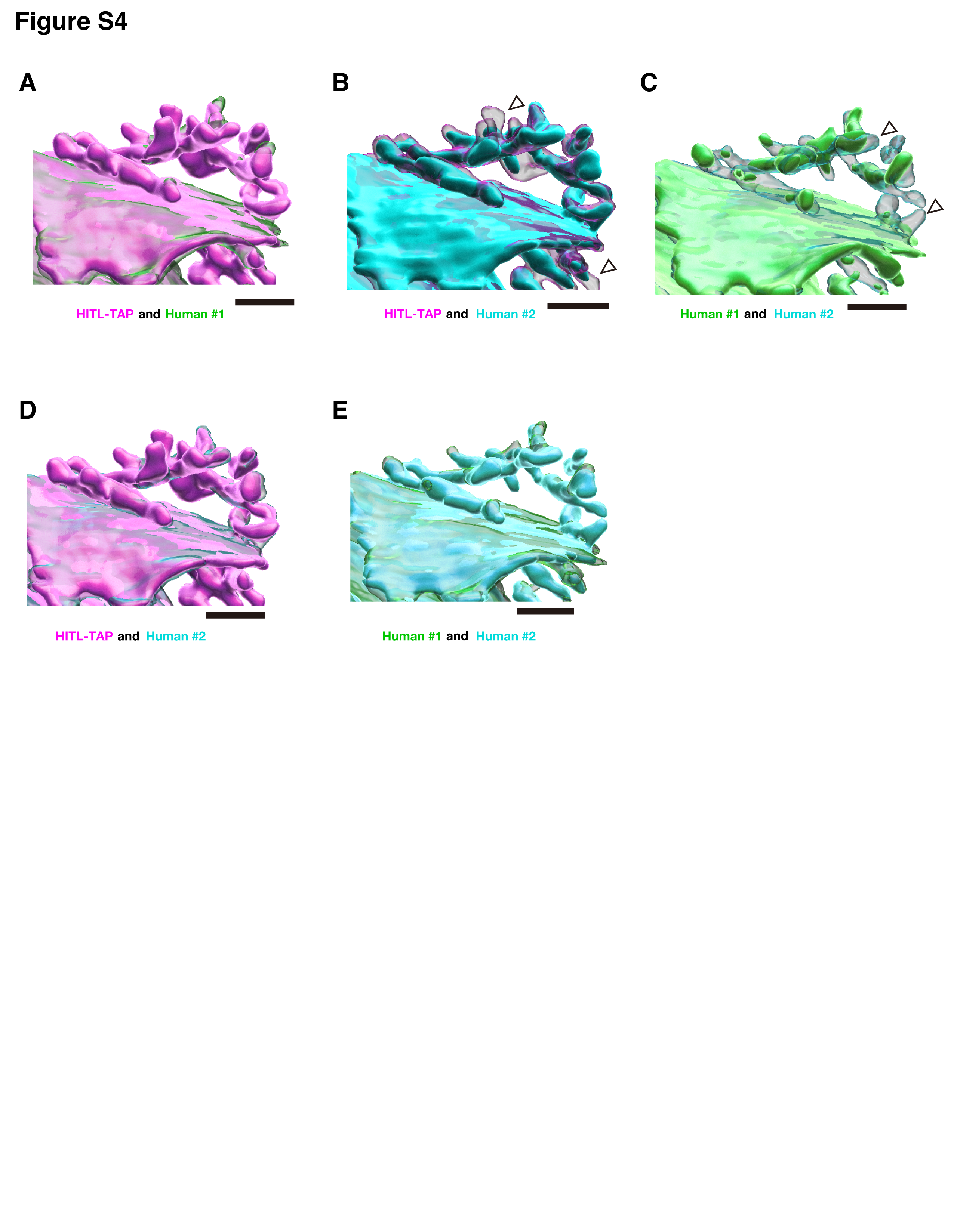

Supplement: S4 Fig — Two of the 3D reconstructions by the HITL-TAP, Human #1 or Human #2 are compared. Note that the human annotators tend to segment lamellar structures more extensively than the HITL-TAP. Also note that the human annotators tend to omit tubular structures, which were recognized by the HITL-TAP (arrowheads). (A) Magenta (filled): HITL-TAP; Green (transparent): Human #1. (B) Cyan (filled): Human #2; Magenta (transparent): HITL-TAP. (C) Green (filled): Human #1; Cyan (transparent): Human #2. (D) Magenta (filled): HITL-TAP; Cyan (transparent): Human #2. (E) Cyan (filled): Human #2; Green (transparent): Human #1. Scale bars, 150 nm. The raw EM data are deposited in the EMPIAR (EMPIAR-11449). EMPIAR, Electron Microscopy Public Image Archive; EM, electron microscopy; 3D, three-dimensional; HITL, human-in-the-loop; TAP, three-axes prediction. (TIF) [file pbio.3002246.s004.tif]

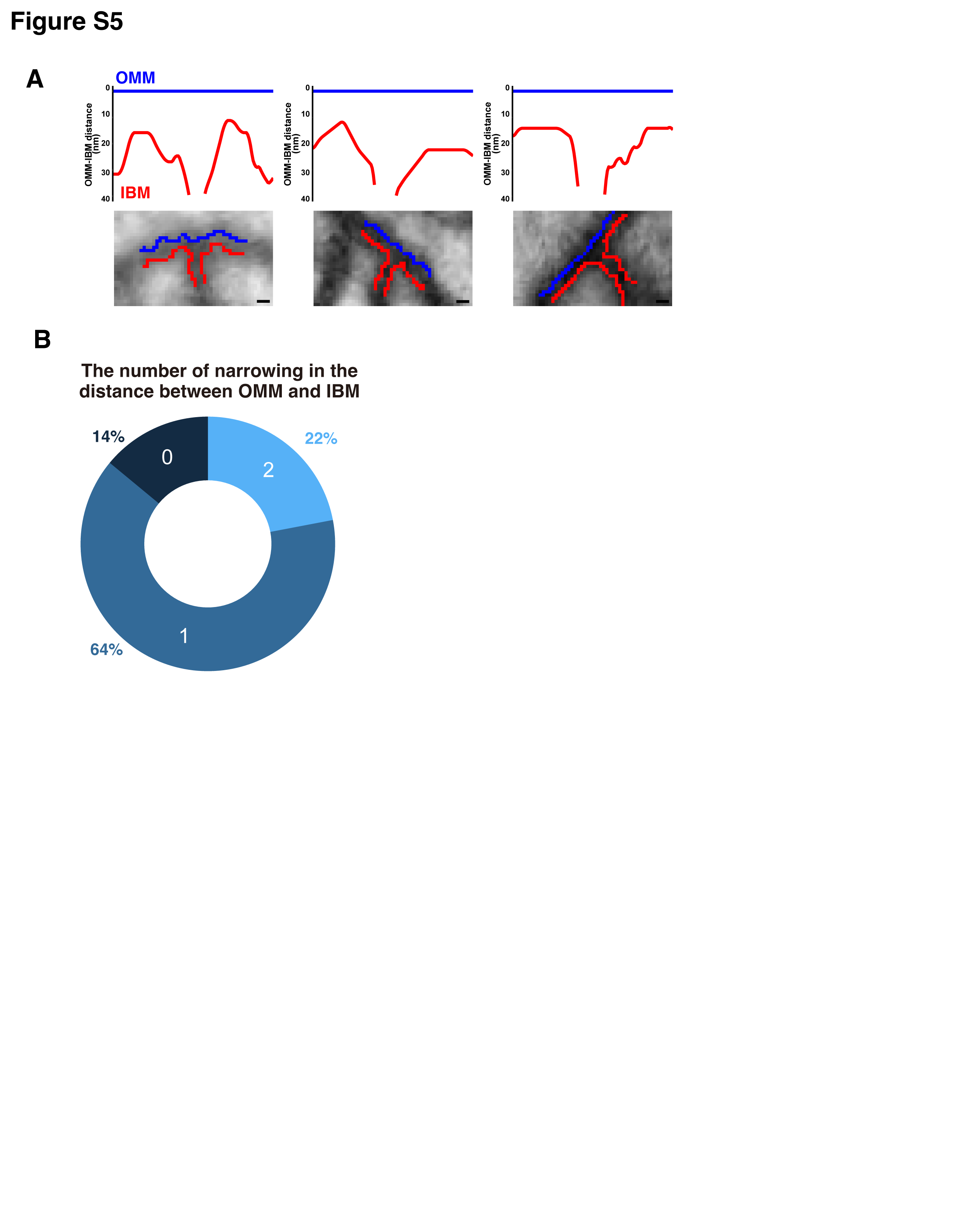

Supplement: S5 Fig — (A) The distance between the OMM (traced by blue line) and IBM (traced by red lines) was defined as the thickness of the membrane at the boundary of mitochondria. Examples of CJs in which the OMM-IBM distance was narrowed bilaterally (left), unilaterally (center), or neither (right) are shown. The graphs represent the OMM-IBM distance measured from the EM images at the bottom. Source data can be found in S15 Data. Scale bars, 20 nm. (B) The percentages of CJs in which the OMM-IBM distance was narrowed bilaterally (2), unilaterally (1), or neither (0) are shown. The raw EM data are deposited in the EMPIAR (EMPIAR-11449). EMPIAR, Electron Microscopy Public Image Archive; OMM, outer mitochondrial membranes; IBM, inner boundary membranes; CJ, crista junction; EM, electron microscopy. (TIF) [file pbio.3002246.s005.tif]

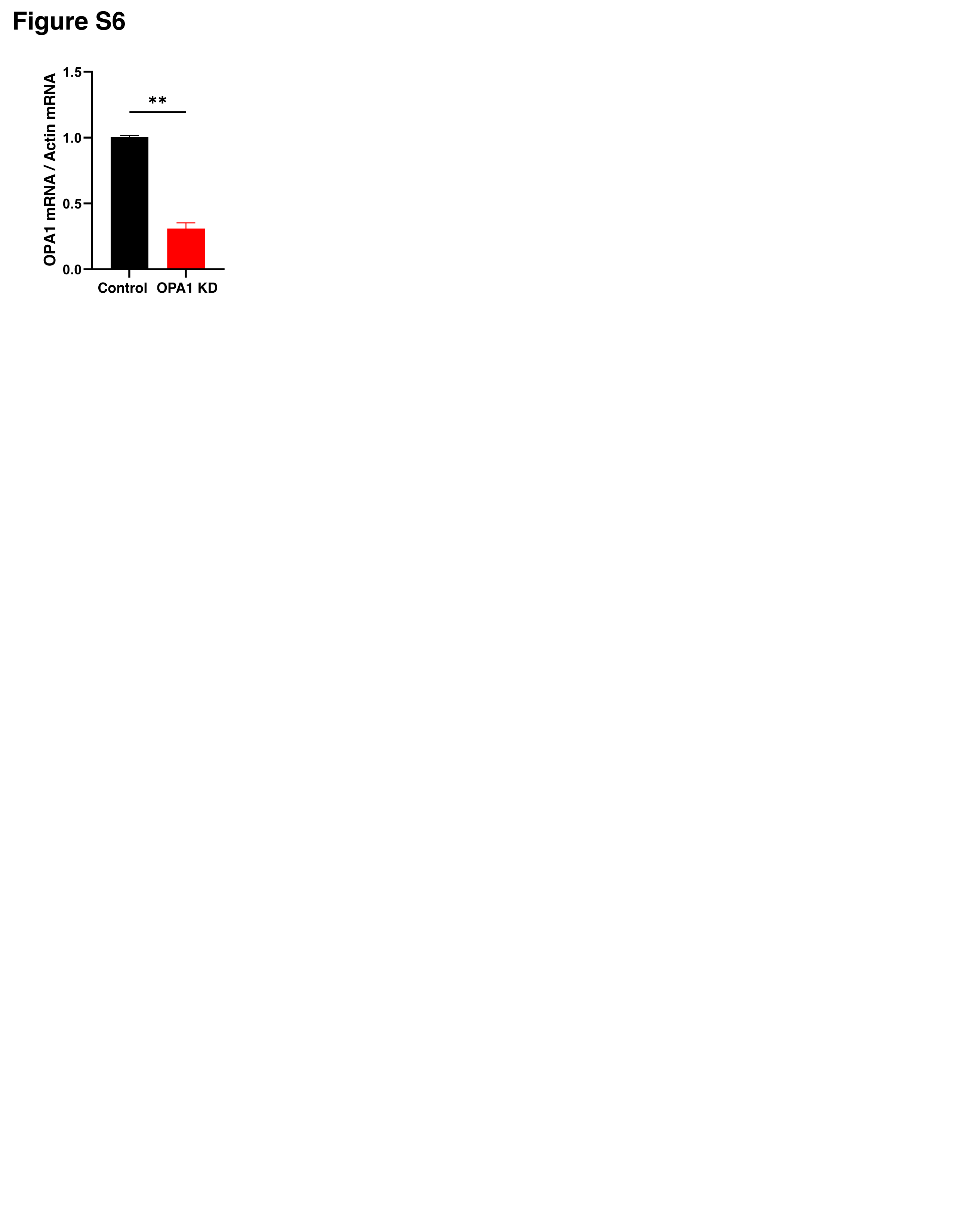

Supplement: S6 Fig — The amounts of OPA1 mRNA were measured by quantitative RT-PCR and normalized by Actin mRNA levels. Error bars indicate standard errors from duplicated samples of quantitative PCR. Source data can be found in S16 Data. **p < 0.01, Student’s t test. The raw EM data are deposited in the EMPIAR (EMPIAR-11449). EMPIAR, Electron Microscopy Public Image Archive; EM, electron microscopy; OPA1, optic atrophy 1. (TIF) [file pbio.3002246.s006.tif]

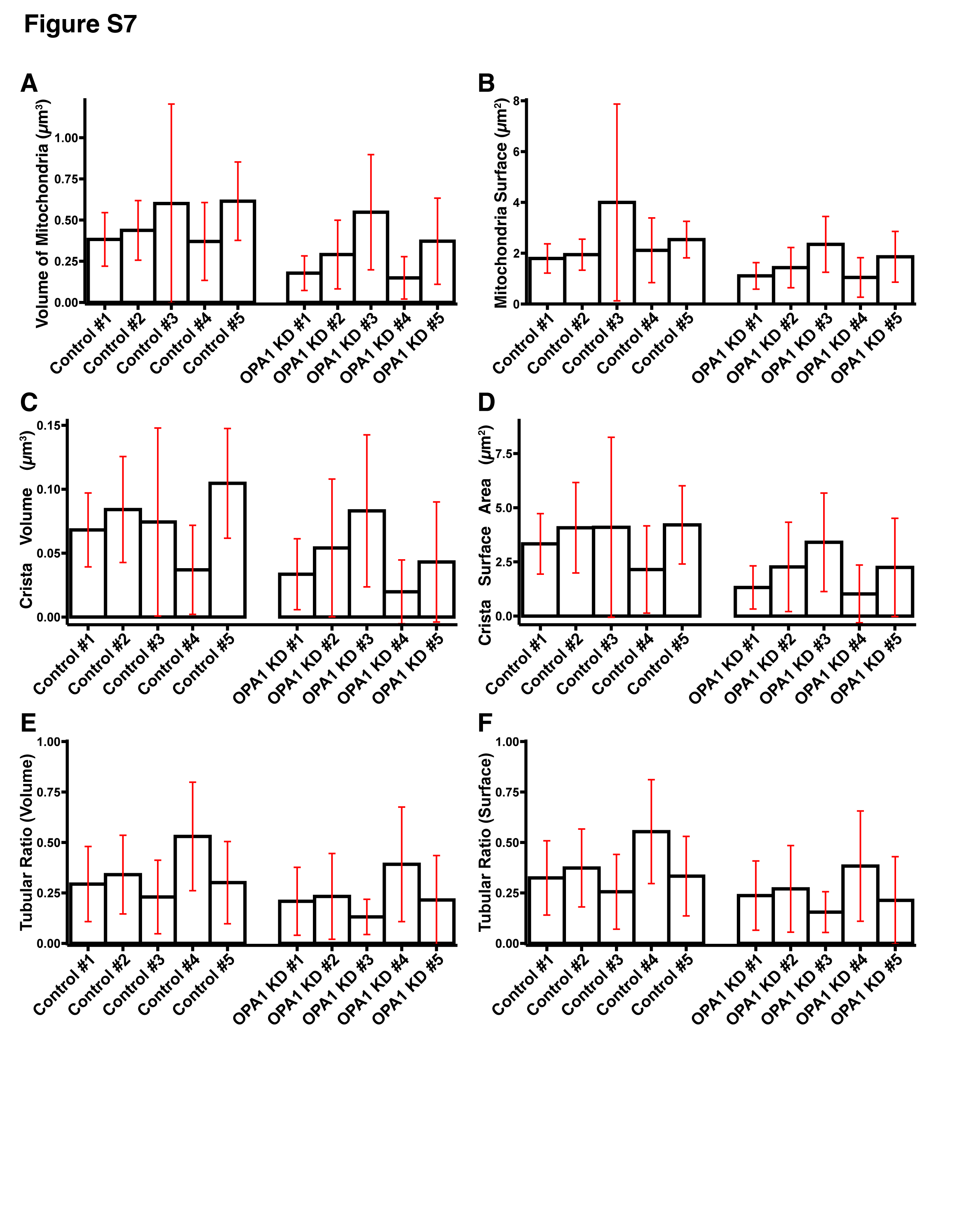

Supplement: S7 Fig — (A–F) Statistical results for each cell in control and OPA1 KD cells. Red lines show mean ± SD. Source data can be found in S17–S22 Data. The raw EM data are deposited in the EMPIAR (EMPIAR-11449). EMPIAR, Electron Microscopy Public Image Archive; EM, electron microscopy; OPA1, optic atrophy 1. (TIF) [file pbio.3002246.s007.tif]

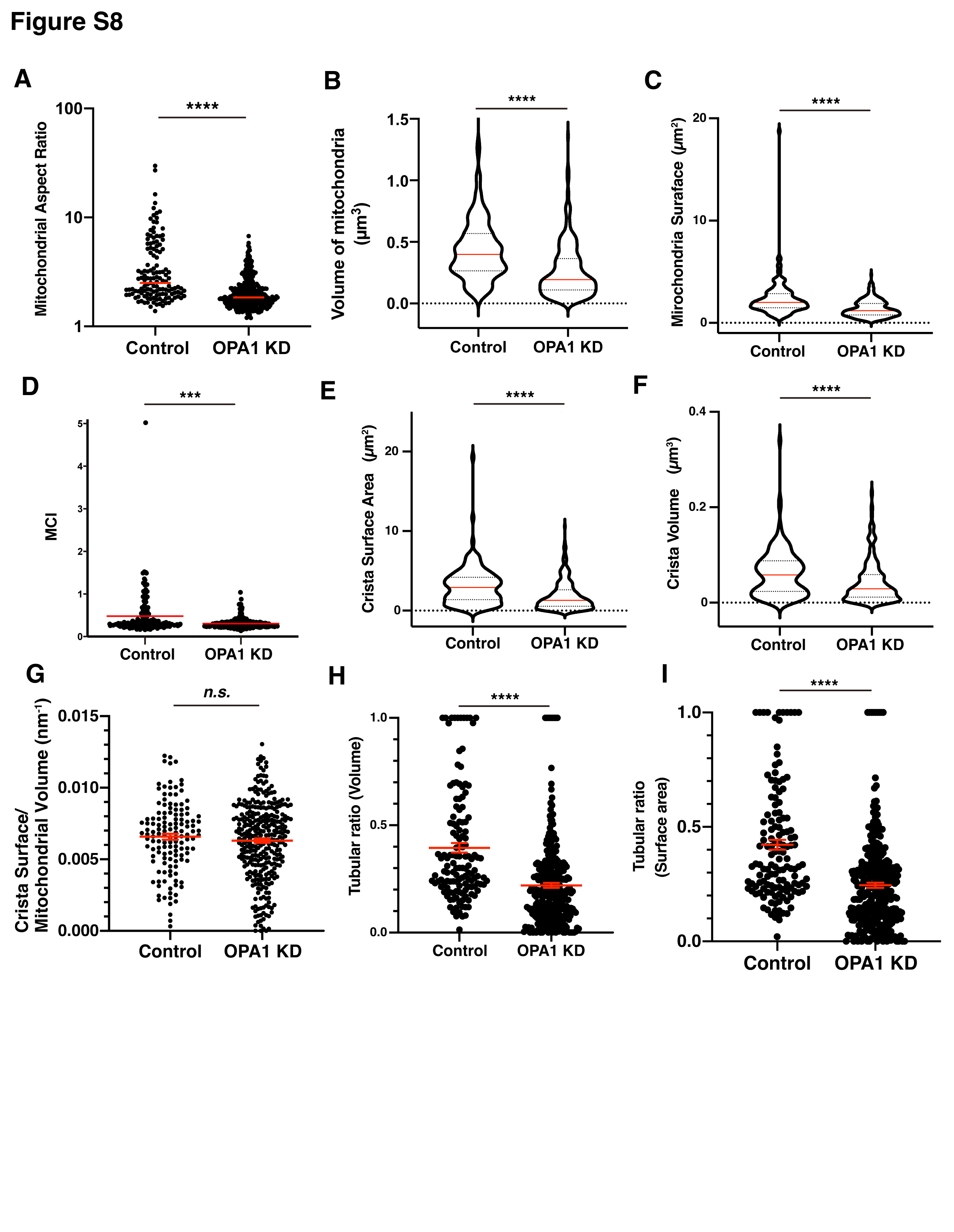

Supplement: S8 Fig — Statistical analyses of the control and OPA1 KD mitochondria and cristae. (A) Maximum length per minimum length is shown with median (red lines). Source data can be found in S23 Data. ****p < 0.0001, Mann–Whitney test. (B–I) Parameters of mitochondria and cristae from the control or OPA1 KD cells are indicated. Red lines show median in (B–F) and mean ± SE in (G–I). Source data can be found in S17–S22, S24 and S25 Data. ***p < 0.001, ****p < 0.0001, Mann–Whitney test. The raw EM data are deposited in the EMPIAR (EMPIAR-11449). EMPIAR, Electron Microscopy Public Image Archive; EM, electron microscopy; OPA1, optic atrophy 1; PCA, principal component analysis; MCI, mitochondrial complexity index. (TIF) [file pbio.3002246.s008.tif]

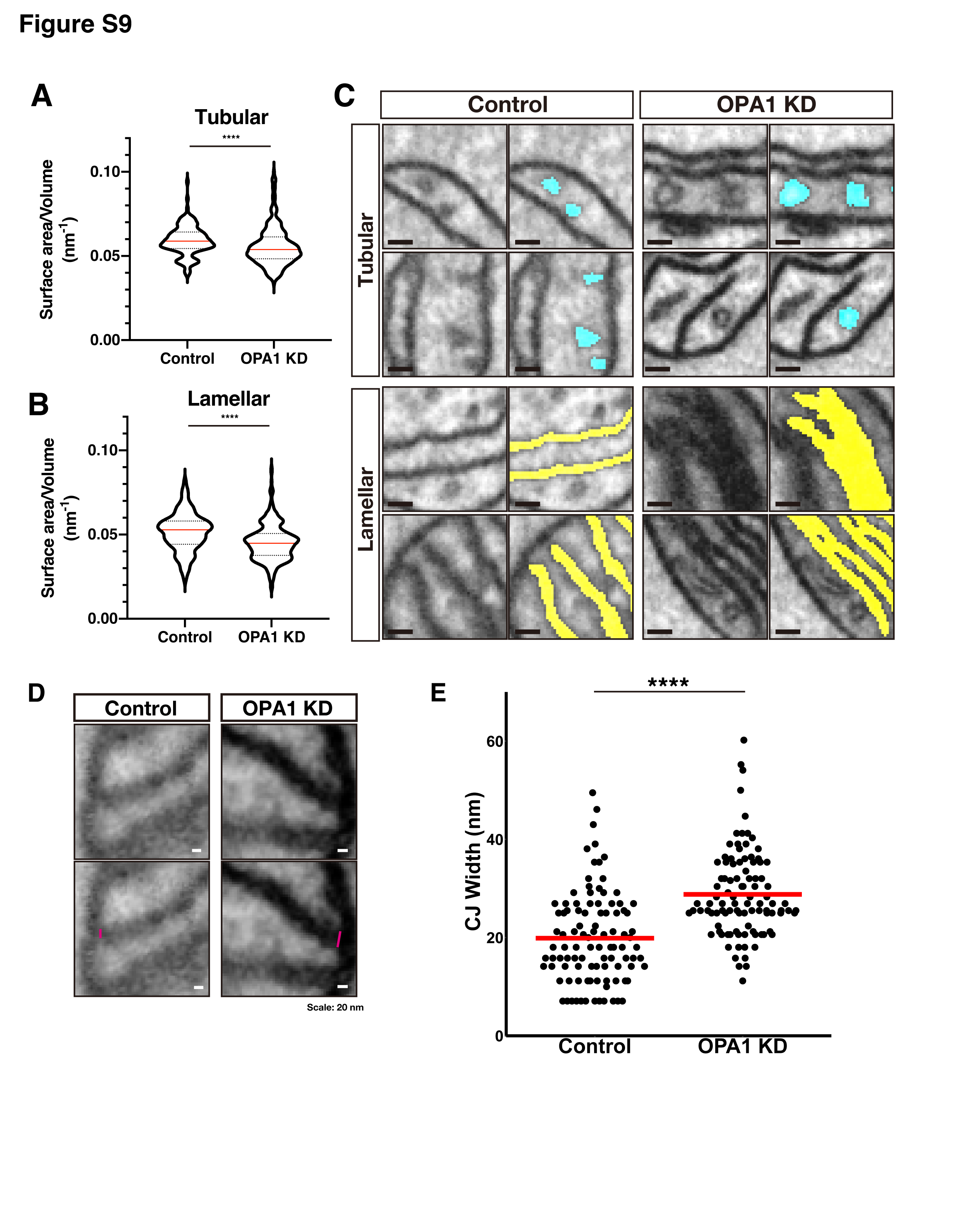

Supplement: S9 Fig — (A, B) Surface area per volume of tubular (A) or lamellar (B) cristae are shown with median. Source data can be found in S26 and S27 Data. ****p < 0.0001, Mann–Whitney test. (C) Representative EM images of tubular and lamellar cristae from the control and OPA1 KD cells. Note that both lamellar and tubular crista structures were thicker in the OPA1 KD mitochondria. Scale bar, 100 nm. (D) Representative CJs from the EM images. The red lines show CJs. (E) The quantification result of CJ width extracted from the EM images with 5 × 5 nm/px resolution. Red lines indicate median. Source data can be found in S28 Data. ****p < 0.0001, Mann–Whitney test. The raw EM data are deposited in the EMPIAR (EMPIAR-11449). EMPIAR, Electron Microscopy Public Image Archive; EM, electron microscopy; OPA1, optic atrophy 1; CJ, crista junction. (TIF) [file pbio.3002246.s009.tif]

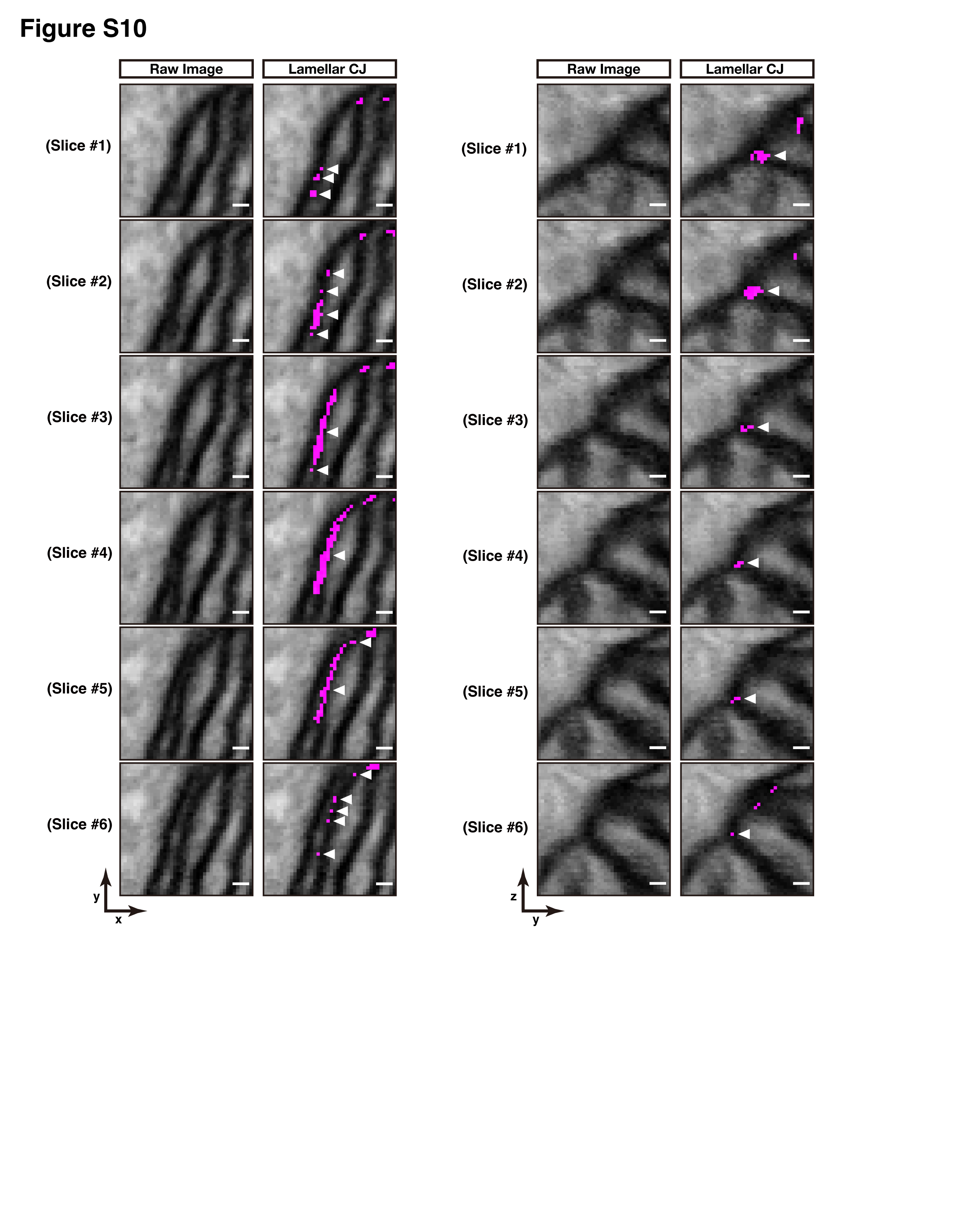

Supplement: S10 Fig — Slot-like CJ was observed in XY plane and YZ plane. Allow heads indicate a single connected slot-like lamellar CJ. Magenta: lamellar CJ. The raw EM data are deposited in the EMPIAR (EMPIAR-11449). EMPIAR, Electron Microscopy Public Image Archive; EM, electron microscopy; CJ, crista junction. (TIF) [file pbio.3002246.s010.tif]

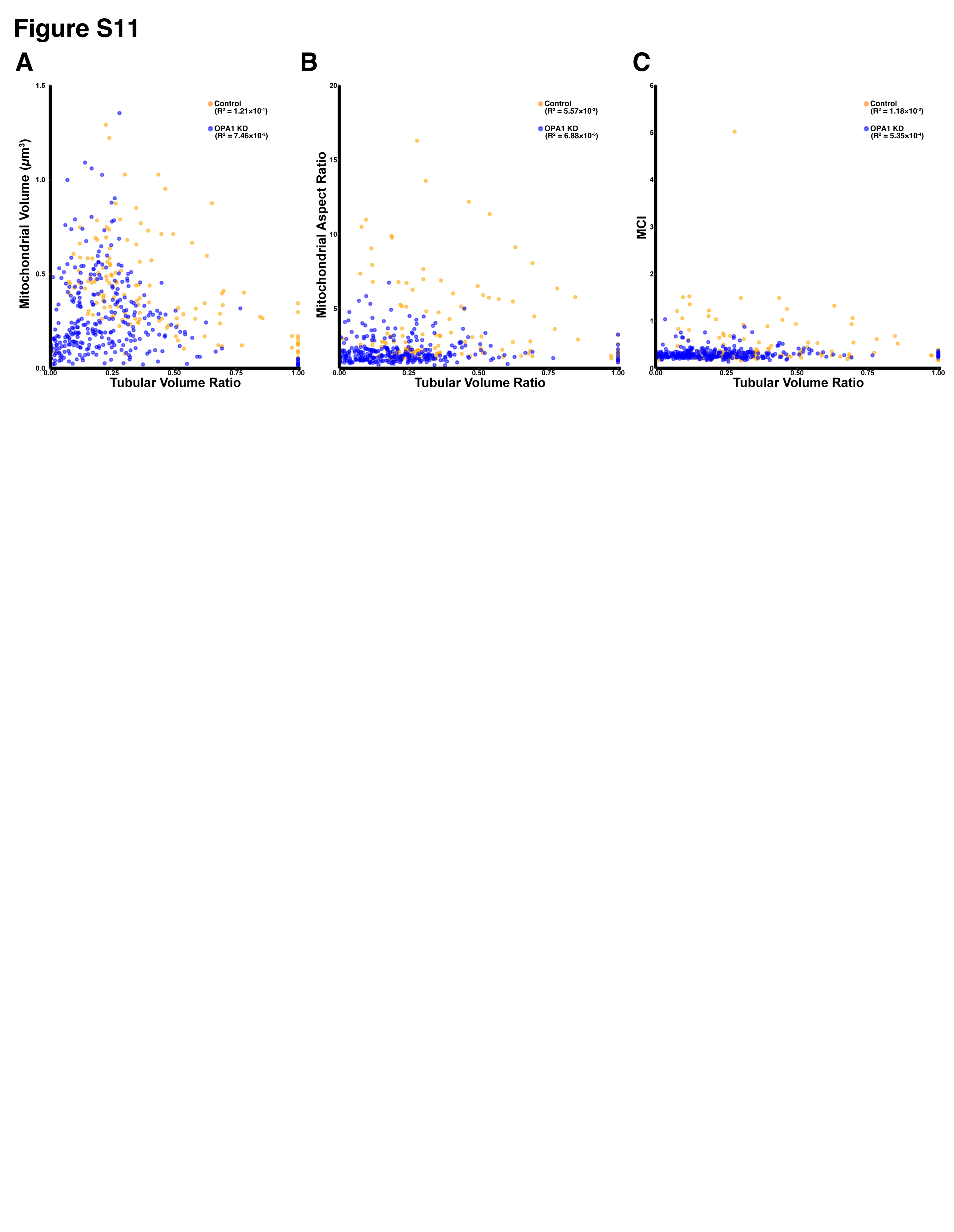

Supplement: S11 Fig — (A–C) Statistical analyses of the tubular crista volume ratio and mitochondrial 3D structure in the control and OPA1 KD mitochondria. R2 indicate the coefficient of determination. Source data can be found in S29–S31 Data. The raw EM data are deposited in the EMPIAR (EMPIAR-11449). EMPIAR, Electron Microscopy Public Image Archive; EM, electron microscopy; OPA1, optic atrophy 1; MCI, mitochondrial complexity index. (TIF) [file pbio.3002246.s011.tif]
