# Supplementary material for: An interactive deep learning-based approach reveals mitochondrial cristae topologies
Source: PLoS Biol. 2023 Aug 31;21(8):e3002246. doi: 10.1371/journal.pbio.3002246 (PMC10470929; doi:10.1371/journal.pbio.3002246)
Supplement: Attachment — Submitted filename: PLOS_Suga Nakamura et al rebuttal#.pdf [file pbio.3002246.s051.pdf]

We really appreciate the comments from the reviewers. Below are the point-by-point responses.

Rev. 1:
The revision by Suga et al., addresses the issues that I raised, but unfortunately brings up another issue in the process of addressing the crista junctions (CJs). I should say that I do appreciate particularly how the authors addressed points about CJ analysis, addressed in Figures 5 and S5 of the revision.

Although I think the revision is much improved after their revision, the issue with the interpretation of the CJs the authors rendered on lamellar cristae must be addressed before acceptance of the paper to PLoS Biology. This and some other major points are listed below, followed by some minor language corrections.

I reiterate that this manuscript reports an important tool for rapid and robust analysis of mitochondrial ultrastructure, revealing that mammalian mitochondria bear tubular and lamellar cristae, whose balance is affected by OPA1. But the potential limitation of FIB-SEM visualization of CJs (affecting PHILLOW processing) I point out below must be addressed.

Major points:

1) The very long, slit-like CJs reported in the aforementioned figures and described in the chapter starting on line 261 contradict observations of CJs rendered from electron tomograms in MEFs and other metazoans. Here the authors observe very long CJs at the base of lamellar cristae. In contrast, in all electron-tomographic observations I know of, the base of lamellar cristae are attached to the inner boundary membrane (IBM) by multiple tubular or in some cases oval CJs that are often adjacent to each other.

The authors should at least acknowledge that these PHILLOW renderings of CJs in lamellar do not agree with tomogram-renderings of the CJs (multiple papers listed in the cited Panek review). Perhaps this is an artifact of the electron beam voltage (stated on line 740), which observes structures ~20 nm deep into the sample, which is more than the 10 nm thick milling sections (line 739). This may result in the appearance of contact along the lamellar crista border with the IBM, when in reality there is a small

gap between parallel crista and IBM membranes in between a string of adjacent tubular/oval CJs along this border. For example, the second crista from left shown in Figure 5C middle panel agrees with tomogram renderings of CJs, but the two most right ones do not.

Indeed, part of our results do not align well with previous reports using electron tomography. We discussed the apparent discrepancies between this study and previous studies in the Discussion (line 480-490).

It is very hard to evaluate the rendered CJ models without any of the raw images of the FIB-SEM section that contains this/these CJs. The authors can also show this data to support their long slit-CJs or demonstrate any underlying limitations for rendering adjacent CJs that can be addressed by future optimization.

Thank you for this suggestion. Images and videos of segmented slot-like CJs and corresponding row images have been added (Supplementary Fig.S10 and movie S7). In conjunction with the newly added discussion, we believe that we have provided ample information for readers to evaluate our results.

This also raises question for me about how CJ numbers were calculated in Fig 7E. Were these long CJs potentially arising from the aforementioned artifact considered a single crista? Since tubular cristae are convincingly shown to be reduced in OPA1 knockdowns (KDs), and these seem to have conventional tubular CJs (Fig 5), could the increase in long CJs of lamellar cristae be true reason for 1) less CJs and/or 2) apparently wider CJs?

If not done so already, I would suggest that CJ measurements be made exclusively on a specific crista type. The tubular cristae may be the best target given the problems with the long CJs of lamellar cristae.

We appreciate this reviewer for pointing this out. We found that the numbers of CJs connected with tubular were decreased by OPA1 KD, while those connected with lamellar cristae did not show significant change. These data are now added in Fig.7 G-H .

2) This and the next point are less major than the previous point. The authors in

several points in the manuscript state that Mic10 and Mic60 ablation results in onion-like cristae (lines 412 and 508). The phenotypes of these two genes are stacked detached cristae often longer in length. Perhaps the onion-like cristae are seen occasionally in these mutants, but this phenotype typifies yeast cells in which ATP synthase dimerization is impaired (e.g. Paumard et al, 2002 EMBO J 21:221-30).

As per this comment, I do not follow the logic presented by the authors from line 413, somehow correlating CJ density and the appearance of onion-like structures in OPA1-silenced cells. I agree with the authors statement that this represents a deformed IM, most likely due to a IM fusion defect. However, I still do not see how this is connected with CJ formation, and would report these data in a different context.

We agree that this rationale was difficult for readers to follow, and therefore we have removed that section from the manuscript.

3) On lines 427-8, the authors write how crista contribute to "biochemical reactions is speculative". True, there is much debate about whether cristae increase the surface area of the mitochondria to give eukaryotes more ATP per gene, but I also think work such as the cited Wolf article (2019, EMBO J) nicely demonstrate the notion these are bioenergetic sub-compartments within the organelle. So I do not think the function of cristae as bioenergetic subcompartments is speculative, but well established by this and other studies showing enrichments of respiratory chain complexes in crista membranes.

Although our original sentence did not mean “the biochemical reactions is speculative” but “how the crista morphology contributes to the reactions is speculative”, we changed the sentence in a more neutral tone.

Minor Language Correction:

Line 126: "less proofreading time"
Line 228: "of algae and trypanosomes"
Line 229: "laborious,"
Line 254: rather use same 3 decimal recall values as in Fig 4C.
Line 262-3: CJ is rather "a narrow ~20 nm attachment point of a crista to the inner boundary membrane (IBM)" They have been also described as 'narrow necks' or

something like this in the literature.
Line 321: rather "cellular respiration"
Line 394: "of Mgm1p"
Line 403: "suggested that"

Thank you very much for pointing these out. We corrected all of the points raised.

Rev. 2:
The authors partially addressed the major concerns, but not successfully. Rather than the volume of individual mitochondria , the aspect ratio and form factors needed to be measured, as aspect ratio and form factor are parameters that quantify shape. Accordingly, the tubular shape of cristae can still be dependent on the change of shape of individual mitochondria induced by OPA1 KD, rather than by OPA1 directly determining tubular shape. With the Drp1 overexpression experiment being missing, this remains an open question. These analysess must be presented in the manuscript (not only in the rebuttal) but using aspect ratio as the parameter to bin and perform correlations and PCA analyses. The minor concers were not addressed: bottleneck can be considered as a synonim to rate limiting. Thus the authors did not perform the change requested. The same issue occurred with the second sentence about orientation.

Thank you very much for the comments. We calculated the aspect ratio and form factor of each mitochondrion and performed PCA. We also examined the correlation between those parameters and the density of tubular cristae. Both analyses showed that there were no correlations between those parameters and tubular ratio. We added this data in Fig.6C-E and Supplementary Fig.S11.
We removed the sentences this reviewer had concerns.
