# Supplementary material for: An interactive deep learning-based approach reveals mitochondrial cristae topologies
Source: PLoS Biol. 2023 Aug 31;21(8):e3002246. doi: 10.1371/journal.pbio.3002246 (PMC10470929; doi:10.1371/journal.pbio.3002246)
Supplement: Attachment — Submitted filename: Suga, Nakamura et al., Rebuttal#.docx [file pbio.3002246.s050.docx]

We would like to thank the three reviewers for their enthusiastic feedback, constructive criticism, and important suggestions to improve our manuscript. In response to the reviewers’ comments, we have reanalyzed some of our data and rewritten the manuscript to address each point raised. Please find below a point-by-point response:

*Reviewers' comments*

*Rev. 1:*

*Powerful volumetric EM approaches such as FIB-SEM have stirred a revolution in cell biology, allowing the resolution of the organelles and even smaller ultrastructure in diverse cells. However, this method is quite demanding in terms of data acquisition and management (e.g. large file sizes) as well as the actual reconstruction of ultrastructures within the cell. The last aspect is plagued by long man hours needed to manually segment structures (i.e. outline the ultrastructure from the background) for reconstruction. Another problem with manual segmentation is biases introduced by the person modeling each of these structures. The manuscript by Suga et al., presents a very exciting and potentially revolutionary way to segment raw FIB-SEM data in a semi-automized way in conjunction with deep learning to facilitate faster and unbiased reconstruction of cristae, numerous ultrastructures that decorate the mitochondrion. This tool, which the authors have named PHILOW, is a very*

*important advance. Remarkably, PHILOW reconstructs cristae from FIB-SEM data in a more precise and reproducible manner than human reconstructors, as also demonstrated in the manuscript.*

*This unbiased approach allowed the authors to reveal a significant population of tubular cristae thus far hidden from view, so far being obscured by the more prominent lamellar cristae. Furthermore, these tubular cristae are depleted upon downregulation of Opa1, a dynamin-like protein (DLP) involved in cristae remodeling and inner membrane fusion.*

*The paper is more or less well written presenting the PHILOW and its implementation in the analysis of Opa1 knockdowns in mouse fibroblasts. Furthermore, the microscopy images are not only informative, but visually appealing. Given the quality of the data and its presentation as well as the significant methodological advance PHILOW represents and the confirmation that tubular cristae are replete in mammalian mitochondria, I feel that the manuscript upon addressing the points below will be suitable for publication in PLoS Biology. I ask for major revision but assume the authors will have data readily available (e.g an elaboration Figure 6B) to address my point about crista junctions below. If this point is addressed, the manuscript truly represents a methodological advance that is worthy of inclusion into PLoS Biology. Thus, I do not think from the implied data that any new experiments need to be performed. Just more data about CJs should be presented among other major*

*points.*

*MAJOR POINTS*

*1) The manuscript seems to be submitted as a Research Article. But in my opinion , it is a 'Methods and Resources' article. Given the significant methodological advance that PHILOW represent, I reiterate that upon addressing points below, the manuscript is suitable for PLoS Biology but as 'Methods and Resources' one to reflect its scope.*

We appreciate this suggestion and will discuss this with the Editor.

*2) The authors claim their method allowed them to measure the "…number of CJs per mitochondria" (lines 418-9) and thus represents "…to our knowledge, this is the first report of CJ numbers and frequency." (lines 425-6 ). Aside from the scatter plot in Figure 6G and a note in the legend of Figure 6B, there is not data about observation of CJs. Given that CJs have thus far not been observed by FIB-SEM as far as I know, this would indeed be a very big advance. The authors should provide some representative images of 3D reconstructions and/or CJs revealed by segmentation on FIB-SEM sections/slices to show how CJs were revealed by PHILOW. This should also be described in Materials and Methods as for other crista ultrastructural features. Finally, the voxel size is stated to be 4.88 nm x 4.88 nm x 10 nm. CJs are about 20-25 nm in diameter on average. Thus, the CJs seem to be on the very detection limits of FIB-SEM. Thus, the authors really should explain how they observed this*

*feature and the average CJ diameters they observed as well as their occurrence. Were CJs seen only on XY axis or also on XZ and YZ as well? If the authors can provide ample evidence for CJ detection using PHILOW, they should bring this aspect out more in front of the manuscript and not have these data buried in the Results section as it currently is, especially given the earlier here quoted claims they make in the Discussion.*

We apologize for the lack of detailed data and explanation about the CJ detection. Representative images from CJs associated with lamellar and tubular cristae are added in Figure 5. We added a section “3D reconstruction of crista junctions from FIB-SEM images” in the main text and “Detection and quantification of crista junctions” in the Method section.

The density and average diameter of CJs are now shown in new Figure 7F, S9.

*3) Related to point 2 above, on lines 290-291 the authors interpret the reported widening of crista lumina as confirmation that OPA1 is involved in CJ constriction. However, since CJ width was not directly assayed in this experiment, this statement is not true. The authors should rephrase this to say their results confirm that OPA1 was a role in narrowing the width of crista lumina or even better, as per point 2, report the measured widths of CJs in this experiment. Once again, I think that the potential capacity of PHILOW to assay CJ properties in a high-throughput way is very exciting.*

We appreciate this reviewer’s suggestion, which is critical for determining the effect of OPA1 KD. We measured the widths of CJs and added the result in Figures S9 D,E. Our results confirm that OPA1 has a role in narrowing the width of CJ.

*4) The final section of the Results (starting on Line 345), the authors compare their results on OPA1 RNAi-silencing in mouse fibroblasts with results reported in other articles in a somewhat misleading way. First of all, discussed references 33, 41 and 42 studied the inner membrane DLP MGM1 in baker's yeast, not OPA1 per se. While these two proteins likely perform the same or overlapping functions in IM fusion and crista maintenance, they may not be true homologs. This issue of course is outside the scope of the paper, but the authors should at least say which organism these data were derived from and mention the yeast name of the protein. Second, the authors write "…OPA1 KD also produces mitochondria with an onion-like structure [33]". The cited reference did not use OPA1 KD as written but a temperature-sensitive mutant of MGM1. AS far as I know when the yeast is put in the non-permissive temperature of 37 ºC, the mitochondrial fusion is inhibited, presumably due to point*

*mutations in the GTPase domain. This is not a knockdown not a knockdown per se. Third, prior to this the term 'Opa1 KD' was used in the context of septum formation. Again, reference 33 uses the temperature-sensitive MGM1 mutant, reference 41 does not mention septa at all, and reference 42 uses a mgm1 knockouts in strains where other DLPs are also deleted. Thus, references 41 and 42 are not appropriate here and in fact the authors state the fzo deletion leads to more septa formation than mgm1 deletion in reference 42. Finally, the authors discuss the phenotype of a site mutation in the C. elegans EAT-1 protein. They name this properly but fail to mention this is site mutation in the GTPase domain and not a RNAi-silencing. Thus, the authors should correctly discuss their OPA1 KDs phenotypes in the context of these various genotypes/conditions/organisms.*

We appreciate that Reviewer 1 pointed out our mistakes in the citations. Indeed, ref. 41 and 42 are not adequate here. We instead cited Hu et al., 2020, which investigated the OPA1 KO MEF.

The temperature-sensitive mutation in mgm1 used in Harner et al., and the mutation in EAT-3 are now described correctly (Line 406-407).

*5) The way the data in the figures in written about in the results is done so in a very confusing way. For example Figure 3D is mentioned before Figure 3C on page 6. Figure 5 is even more confusingly presented, with 5F mentioned first and then 5A. Furthermore, the following main figures are never referenced in the manuscript: Fig 2E, 2F , 6A and 6B. The authors should order their figures in the order they are mentioned in the manuscript and mention ALL figure elements. Please also treat the supplemental Figures the same way.*

We apologize for the confusing structure of the manuscript. By adding a chapter describing CJ quantification, we rearranged the structure of the manuscript. Now the figures are rearranged in the order they appear in the text.

*6) Data in 6A can go into supplement as it only verifies OPA1 RNAi and thus is a control. Figure 6B notes that the CJ area is less but not indicated at all. This refers to Point 2 above. The CJs should be highlighted and the sections with CJs of these images should be shown, as also mentioned in point 2.*

Former Fig. 6A is now moved to Supplementary Fig. 6.

Also please see the response to points 2 and 3.

*7) Tables 1-4 are not contain any descriptive titles or small legend.*

Titles are added to the tables.

*8) I could not open Videos 1,2 and 4 as there were issues with not having the proper codec. I did not have such issues with the other videos. Please be sure Videos 1,2 and 4 use the same codec as Videos 3 and 5.*

We apologize for the inconvenience. We coded the videos compatible with both Windows OS and Mac OS.

*9) The paper seems to focus mostly on the application of PHILOW on mitochondria with tubular and lamellar cristae. However, FIB-SEM has been used to observe the unique cristae of algae (Uwizeye et al., 2021 PNAS 118 (27): e2025252118) and trypanosomes (Bily et al., 2020 J Euk Micro 68: e12846). The authors should at least mention whether they think PHILOW will work on other crista shapes, especially hard to discern ones sometimes found in such organisms.*

Thank you for pointing out these papers. Now the segmentations in these papers are discussed in the result sections (Line 226-230).

*10) Lines 258-61. Authors assay 5 cells from control and OPA1 KD, observing 135 and 324 mitochondria respectively. I would appreciate if the authors had a plot indicating average mitochondria per cell with standard deviation.*

We added a new Supplementary Fig. 7, which shows the quantification results per cell with standard deviation.

*11) What was the rationale of the authors reporting surface area/volume of mitochondria (Lines 264-7, Table 1)?*

The surface area/volume of mitochondria has been used as an index for the complexity of mitochondria structure. Indeed both Uwizeye et al. and Bily et al. have also used this value as an index for the complexity of mitochondria. We clarified this in the main text (Lines 303-304).

*12) Pertaining to point 2 above. According to the methods, the the acquired FIB-SEM data had a final voxel size of 4.88 nm x 4.88 nm x 10 nm (line 686). Why is the z axis different? Is this really isotropic?*

For most of our analyses, the voxels were binned in x-y planes. Therefore, the voxel size becomes 9.76×9.76×10 nm^3^. This dimension practically worked as isotropic in our analyses.

*Minor points:*

*1) Abbreviations in Figures should be defined in their respective legends (e.g. IoU).*

Abbreviations are added in each figure legend.

*2) Lines 2675-8: "…a gene responsible for optic atrophy." Citation missing.*

We apologize for missing the citation. Citations *Delettre C et al.,* and *Alexander C et al* are now moved after this sentence.

*3) Line 265, should read "…mean mitochondrial surface area…"*

The sentence is corrected accordingly.

*4) Throughout text, if used as adjective, use singular form 'crista' (e.g. lines 383, 395)*

“Cristae” used as an adjective were replaced with crista.

*5) In Methods, delete 'for' in e.g. "…for 3 times…" (line 657). Also, please either use numerals or words for numbers but do not mix these 2 forms.*

We corrected this point.

*6) Line 720, should read "…with the following parameters:"*

We corrected this point.

*Rev. 2:*

*The manuscript by Suga, Nakamura et al report a novel image analysis method for FIB-SEM micrographs that decrease processing time, named PHILOW. This new method allowed for a higher throughput 3D reconstruction of mitochondrial cristae, segmenting in a manner that could even supersede the capacity of the human eye. Using this method, they conclude that an underappreciated defect caused by OPA1 knock down is a change in cristae orientation, as well as in the ratio of lamellar and tubular cristae. In addition, authors claim that this is the first time reporting total cristae junction numbers and frequency. The method seems robust and provides a significant advancement in terms of speed and segmentation. In addition, authors made it publicly available, which is great. However, the main concerns related to the interpretation of the novel biology and roles identified for OPA1, as well as the interpretation of the authors about the role of lamellar versus tubular cristae.*

*1) Most of the data shown should be binned by individual mitochondria, as there is a possibility that differences in mitochondrial size are driving most of the phenotypes. Authors should show whether there is any correlation between number and frequency of cristae junctions, lamellar and tubular mitochondria with mitochondrial aspect ratio, form factor or size. Among the heterogenous structures of control mitochondria (Figure 5B), one could see that there is a correlation between of how the cristae are organized depending on the shape of the individual. Therefore, is OPA1 changing these novel parameters because it is inducing fragmentation or because it is truly reflecting a specific action of OPA1 in the cristae.*

To clarify the PCA and hierarchical analysis, small mitochondria are defined in Figure (A) and the ratio of tubular cristae in those mitochondria were investigated (B). We find that the reduction of tubular cristae is still observed in these small mitochondria populations. Furthermore, both in the control and OPA1 KD cells, the tubular ratio was not correlated with the mitochondrial volume as shown in (C). These strongly suggest that the morphological changes of cristae in OPA1 KD cells is not because of the fragmentation.

Also, in the new manuscript, CJ density is incorporated into the hierarchical and principal component analyses, revealing a low correlation between them. The new PCA and hierarchical analysis still show that the cristae density, mitochondria size and tubular ratio are not well correlated. New sentences describing these notions are added (Lines 344-349).

*2) Authors should analyze cristae structures using their new method in cells with shorter mitochondria without inducing OPA1 cleavage (i.e. Drp1 overexpression). Such an experiment is required to prove the authors conclusions of OPA1 being responsible for controlling these new aspects of mitochondria cristae.*

As answered above, there is a low correlation between the size of mitochondria and tubular ratio, both in the control and OPA1 KD mitochondria. Furthermore, the ratio of tubular cristae was decreased by OPA1 KD even among small mitochondria (please see the response to point 1). We believe that this point is well addressed by the above analyses.

*3) Authors claimed that they could count for the first time the number of CJ. Could they quantify mitochondrial contact sites as well (area where the distance between the outer and inner membrane is reduced)?*

Thank you for this suggestion. We examined the mitochondrial contact sites from a randomly selected set of CJs, of which 86% displayed a narrowing in the distance between the OMM and IMM on at least one side. This result is now included in the new Figure S5.

*Minor comments: the paper include inaccurate statements such as:*

*1) "Since diffusion of ions and metabolites through the narrow compartment of cristae represents a significant rate-limiting step of biochemical reactions in mitochondria" This is not true, diffusion of ions is not the rate limiting step. In the case of OXPHOS, is ADP availability in the matrix.*

Thank you for pointing this out. We revised the sentence as follows.

"Since the narrow compartment of cristae represents a significant bottleneck for the diffusion of ions and metabolites that are required for biochemical reactions in mitochondria" (lines 61-63).

*2) "Since the orientation of cristae directly affects the distance metabolites need to travel from the cytosol to biochemical reaction sites on IMM, this finding raises the possibility that cristae orientation is regulated to meet metabolic needs of ever-changing cellular conditions." There are many inaccuracies: a)orientation does not directly affect distance, b) it could be cristae length and distance to the outer membrane and c) how orientation of the cristae is the best way to regulate ever changing conditions. I would strongly encourage the authors to remove this sentence.*

We agree with the point. Therefore, we revised the sentence as follows.

“Since the orientation of cristae affects the crista length, this finding raises the possibility that cristae orientation is regulated to meet metabolic needs of ever-changing cellular conditions by adjusting the distance metabolites need to travel from the cytosol to biochemical reaction sites.” (lines 492-496).

*Rev. 3:*

*The paper proposed a human-in-the-loop object segmentation approach to study mitochondrial cristae topologies. My comments are concerned with the deep learning part of the manuscript. I can't judge the biological significance of the finding.*

*The proposed method has three key elements:*

*1. A 2d UNet++ for predicting segmentations*

*2. Human-in-the-loop for prediction correction and adding training*

*3. A 3D integration step to improve robustness*

*The authors demonstrated impressive F1-score performance.*

*Comments*

*1. There are very few details about the deep learning methods. More details on the training/test/validation data are needed to better understand the performance.*

We apologize for the lack of detailed information.

We have added details of evaluation processes of HITL as follows.

“When TAP was applied to segment mitochondria from a 10×10×10 nm isotropic volume (309×732×1554 voxels, 10 out of 309 slices were used for training and all 309 slices were applied for test), strikingly, the variance of intersection over union (IoU) between neighboring xy-slices of inferences was ten times lower compared to that without TAP (0.0082 and 0.0889 with or without TAP, respectively) and three times lower than manual segmentation (0.0237) (**Fig. 2B**).” (lines 144-150)

“To test the HITL scheme, we segmented mitochondria from 248×1147×960 voxels FIB-SEM images of NIH3T3 cells at 10×10×10 nm per voxel.” (lines 162-164)

“Another round of iteration with four more (a total of 10 out of 248) areas of the HITL-learning generated a DL model covering mitochondrial features highly comprehensively. The F1 score compared to the ground truth (GT) was calculated for the predicted results for entire slices, including the slices used as the training data, and reached 0.97 with the constant increase along three iterative cycles (0.906 after the first cycle and 0.924 after the second cycle), whereas a model generated from random ten training areas exhibited a much lower F1 score (0.909).” (lines 171-178)

“HITL iterations were performed on a 93×109×115 voxels volume, and a total of 7 slices out of 93 slices were used to train the model to predict all 93 slices.” (lines 239-241)

*2. Does every human-in-the-loop cycle need to retrain or fine-tune the model?*

For each human-in-the-loop iteration, training data is added and retrained. Since it was not clearly mentioned, we revised the sentence as follows.

“The corrected sections were then incorporated as additional training data to the initial three training data sections for retraining the model (second prediction, **Fig. 3B**).” (lines 168-170)

*3. What do you mean by a probability of 127 or [1, 126]?*

Thank you for pointing this out. Since this did not mean a probability but a voxel value after converting from the range [0, 1] to [0, 255] (uint8), we replaced it with the sentence below.

“Voxels with values exceeding 127 were then annotated as mitochondria, and voxels with [1, 126] values were indicated as low confidence.” (lines 803-805)

*4. It is not clear if any of the F1-score is based on test data.*

We apologize for not stating what data each F1 score is based on. We added it to the main text and figure legends.

“The F1 score of each crop is shown at the right bottom.”

See also our reply to your comment 1.

*5. How robust is the method against different human proofreaders? The authors showed two, and some nontrivial differences can already be observed.*

For the segmentation of the mitochondria, the F1 scores were 0.97 after proofreading by different experts using the HITL+TAP method on a dataset of 240 x 640 x 640 voxels. Furthermore, another additional expert also examined whether the HITL-TAP method reduces human work time by examining the time required for manual, DL baseline, HITL, and HITL-TAP, and confirmed that HITL-TAP is the fastest way to complete reconstructions (**Supplementary Fig. 3**). These suggest that the method is sufficiently robust in the mitochondrial segmentation.

As for cristae segmentation, which showed differences among two human annotators, we did not proofread the prediction results of the model, but only check for any major errors, because the output of the model was highly accurate. Indeed, the differences between the prediction by the model and the segmentation by each of the experts were smaller than the difference between the segmentations by the two experts. Therefore, we believe that the model we created and this method are sufficiently robust. Since we did not specify that no human modification was performed in the segmentation of crista, we added the following sentences.

“These results show that our HITL-TAP method achieved superhuman accuracy in segmenting both lamellar and tubular crista structures and did not require further human proofreading.” (lines 258-260)

“In reconstructing crista structure, the HITL-TAP provided us with highly accurate segmentations of crista without human proofreading. Therefore, the predictions of the model developed after running several HITL iterations were not corrected by humans, but only checked by two experts to make sure that there were no major errors, which were used as the final result.” (lines 823-828)

*6. Segmenting objects from EM images has been a task attempted by many deep learning approaches. The authors cited a few applied to connectomes. No comparison with those existing methods are showed.*

We apologize for the inadequate description of other organelle segmentation approaches. Our method is an approach to build models with less training data, and it is difficult to make a direct comparison as to accuracy with other approaches that require a lot of annotation, especially with 3D U-Net, but we mentioned those approaches in the discussion.

“To perform segmentation on 3D volume datasets of EM and fluorescence microscope images, DL algorithms using information from multiple slices, such as 2.5D U-Net [47] and 3D U-Net [48-51], have been developed and various types of semantic segmentation such as organelles have been investigated. However, creating 3D annotated training data is a very labor-intensive work, as it involves repeatedly annotating tens to hundreds of consecutive slices. While the HITL-TAP on PHILOW and 2D UNet++ has the advantage of quickly creating a high-performing model with fewer slices (10 or less slices in most cases), PHILOW can in principle be combined with any other DL algorithm. Therefore, once a certain volume of proofreading is completed, it can be combined with 2.5D and 3D U-Net to achieve even higher performance and contribute to the reconstruction of complex 3D structures like the ER or Golgi apparatus. The method developed in this study can be applied not only to any EM image analysis, but also to other types of image segmentation and detection.” (lines 463-476)
